# The complete chloroplast genome of *Epimedium wushanense* T. S. Ying. (Berberidaceae), a traditional Chinese medicinal herb

**DOI:** 10.1080/23802359.2020.1715877

**Published:** 2020-01-24

**Authors:** Ting Liu, Xiang Liu, Yanjiao Luo, Yu Yao, Chunmei Wen, Qianru Yang, Guoan Shen, Baolin Guo

**Affiliations:** a College of Traditional Chinese Medicine, Shanxi University of Chinese Medicine, Jinzhong, China;; b Institute of Medicinal Plant Development, Chinese Academy of Medical Science, Peking Union Medical College, Beijing, China;; c Chongqing Academy of Chinese Materia Medica, Chongqing, China;; d College of pharmacy, Shanxi Medical University, Taiyuan, China;; e School of Pharmacy, Jiangxi University of Traditional Chinese Medicine, Nanchang, China

**Keywords:** Chloroplast genome, *Epimedium wushanense*, Berberidaceae

## Abstract

*Epimedium wushanense* is a well-known medicinal plant in Berberidaceae in China. In this study, we sequenced the complete chloroplast (cp) genome of *E. wushanense*. The results showed that the cp genome of *E. wushanense* was 157,283 bp in length, which is composed of a large single-copy region (LSC, 88,579 bp) and a small single-copy region (SSC, 17,082 bp) that were separated by a pair of inverted repeat regions (IRa and IRb, 25,811 bp). The chloroplast genome of *E. wushanense* contains 112 unique genes, of which are 78 protein-coding genes, 30 tRNA genes, and 4 rRNA genes. The overall GC content was 38.78%. The phylogenetic tree analysis showed that *E. wushanense* was closely related to *E. pseudowushanense*, *E. lishihchenii,* and *E. sagittatum*.

*Epimedium wushanense* is a well-known medicinal plant in Berberidaceae in China. The leaves of *E. wushanense* have been officially adopted in the Chinese Pharmacopoeia Commission (2015) under the crude drug names of “Wushan Yinyanghuo” in Chinese. Epimedii Folium has the therapeutic functions of strengthening muscles and bones, dispelling wind dampness and so on. In recent years, with the deepening of the research on the extract of Epimedii Folium, it has been found that Epimedii Folium has more effects on human health, such as treating osteoporosis, improving immunity and anti-aging. In addition, Epimedii Folium extract has also been widely used in cosmetics and health products. For a long time, *E. wushanense* mostly relies on wild harvested resources (Ma et al. 2011; Tong et al. 2013). However, it is hard to clearly distinguish *E. wushanense* from other closely related species in the *Epimedium* family. Herein, we reported the complete chloroplast (cp) genome sequence of *E. wushanense* and revealed its phylogenetic relationships with related species in the Berberidaceae.

In this study, fresh leaves of *E. wushanense* were sampled from the Badong County of Hubei Province, China (N31°09′, E110°27′). Meanwhile, the voucher samples (0434) were collected and deposited at the Herbarium of the Institute of Medicinal Plant (IMPLAD), Beijing, China. Genomic DNA was extracted from the leaves using the improved CTAB method (Doyle 1987). Total DNA was used for the shotgun library construction. The library was sequenced on an Illumina Novaseq PE150 platform and 150 bp paired-end reads were generated. The clean reads were assembled using the program GetOrganelle v1.6.2 (Jin et al. 2018) with the reference chloroplast genome of *E. acuminatum* (GenBank accession number: NC_029941). The gene models were annotated using the online program CPGAVAS2 (Shi et al. 2019) and GeSeq (Tillich et al. 2017), followed by manual correction. The new annotated cp genome of *E. wushanense* had been submitted to NCBI database (accession number: MN857417).

The complete cp genome of *E. wushanense* is 157,283 bp in length and has a typical quadripartite structure, consisting of a large single-copy (LSC) region of 88,579 bp, a small single-copy (SSC) region of 17,082 bp, and two inverted repeat regions (IRa and IRb) of 25,811 bp. The GC content of complete chloroplast genome, LSC, SSC, and IR regions is 38.78, 37.38, 32.75, 43.19%, respectively. The complete cp genome contains 112 unique genes, of which are 78 protein-coding genes, 30 tRNA, 4 rRNA genes. The genes duplicate in the IR region, which are five protein-coding genes (*ndhB*, *rpI23*, *rps7*, *ycf2,* and *rps12*), seven tRNA genes (*trnI*-*CAU*, *trnI*-*GAU*, *trnL*-*CAA*, *trnN*-*GUU*, *trnR*-*ACG*, *trnV*-*GAC*, and *trnA*-*UGC*) and four rRNA genes (*rrn16*, *rrn23*, *rrn4.5*, and *rrn5*). Among all genes, 15 genes contained one intron and three genes contained a pair of introns.

To investigate the phylogenetic relationship of *E. wushanense*, a total of 19 complete cp genome sequences from related species in Berberidaceae was downloaded from the NCBI database. After aligning using MEGA7 (Kumar et al. 2016), RAxML GUI1.5b2 (Stamatakis 2014) was used to reconstruct a Maximum-Likelihood (ML) phylogenetic tree, with *Arabidopsis thaliana* as the outgroup (Figure 1). As a result, phylogenetic analysis indicated that *E. wushanense* is closely related to *E*. *pseudowushanense*, *E. lishihchenii, and E. sagittatum*. The complete cp genome of *E. wushanense* would be a useful resource for studies on population genetics, molecular ecology, molecular identification, and evolution of *Epimedium* and related plant groups.

**Figure 1.**
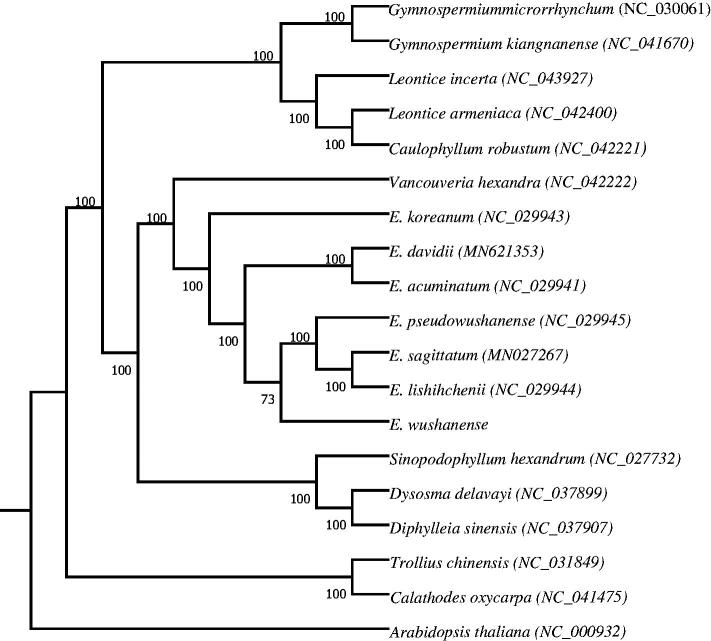
Maximum-likelihood (ML) phylogenetic tree based on complete chloroplast genomes of 19 species, with *Arabidopsis thaliana* as the out group. Numbers above the lines represent ML bootstrap values.
